# Generation and characterization of a knock-in mouse model for Spastic Tetraplegia, Thin Corpus Callosum, and Progressive Microcephaly (SPATCCM)

**DOI:** 10.21203/rs.3.rs-2839029/v1

**Published:** 2023-04-24

**Authors:** Megan L. Ratz, Greg Leary, Andrea Grindeland, Derek Silvius, Joseph Guter, Michael P. Kavanaugh, Teresa M. Gunn

**Affiliations:** McLaughlin Research Institute; McLaughlin Research Institute; McLaughlin Research Institute; McLaughlin Research Institute; McLaughlin Research Institute; University of Montana; McLaughlin Research Institute

**Keywords:** spastic tetraplegia, thin corpus callosum, progressive microcephaly (SPATCCM) mouse model, SLC1A4, ASCT1

## Abstract

SLC1A4 (solute carrier family 1 member 4, also referred to as ASCT1, Alanine/Serine/Cysteine/Threonine-preferring Transporter 1) is a sodium-dependent neutral amino acid transporter. It is highly expressed in many tissues, including the brain, where it is expressed primarily on astrocytes and plays key roles in neuronal differentiation and development, maintaining neurotransmitter homeostasis, and N-methyl-D-aspartate (NMDA) neurotransmission, through regulation of L- and D-serine. Mutations in *SLC1A4* are associated with the rare autosomal recessive neurodevelopmental disorder spastic tetraplegia, thin corpus callosum, and progressive microcephaly (SPATCCM, OMIM 616657). Psychomotor development and speech are significantly impaired in these patients, and many develop seizures. We generated and characterized a knock-in mouse model for the most common mutant allele, which results in a single amino acid change (p.Glu256Lys, or E256K). Homozygous mutants had increased D-serine uptake in the brain, microcephaly, and thin corpus callosum and cortex layer 1. While p.E256K homozygotes showed some significant differences in exploratory behavior relative to wildtype mice, their performance in assays for motor coordination, endurance, learning, and memory was normal, and they showed no significant differences in long-term potentiation. Taken together, these results indicate that some aspects of SLC1A4 function in brain development are conserved between mice and humans, but the impact of the p.E256K mutation on cognition and motor function is minimal in mice.

## Introduction

SLC1A4 (solute carrier family 1 member 4, also referred to as Alanine/Serine/Cysteine/Threonine-preferring Transporter 1, ASCT1) is a sodium-dependent neutral amino acid transporter for L- and D-serine, L-alanine, L-cysteine, L-threonine, L-asparagine and L-valine (Foster et al., 2016; Freidman et al., 2020; Kanai et al., 2013). It is highly expressed in skeletal muscle, lung, kidneys, ovary, heart, intestine, and the brain. In the brain, *SLC1A4* is expressed by astrocytes and plays a key role in NMDA neurotransmission through regulation of D-serine, as well as generally maintaining neurotransmitter homeostasis (Arriza et al., 1993; Foster et al., 2016; Kaplan et al., 2018; Yamamoto et al., 2003). Mutations in *SLC1A4* are associated with spastic tetraplegia, thin corpus callosum, and progressive microcephaly (SPATCCM, OMIM 616657) and sometimes seizures (Abdelrahman et al., 2019; Conroy et al., 2016; Damseh et al., 2015; Heimer et al., 2015; Pironti et al., 2018; Sarigecili et al., 2022; Sedláčková et al., 2021; Srour et al., 2015). This autosomal recessive neurodevelopmental disorder has predominantly been reported in patients of Ashkenazi-Jewish descent, with rare cases in other ethnic populations. The most common mutation alters the glutamate at amino acid position 256 to a lysine (p.E256K) and has a carrier frequency of up to 6% in the Ashkenazi-Jewish population (Damseh et al., 2015; Srour et al., 2015). Most patients are unable to achieve independent walking or speech. Some also have seizures, decreased myelination, and/or brain atrophy. A handful of patients have been identified with mutations that result in frameshift and are expected to result in SLC1A4 deficiency, but missense mutations are more common. Disease symptoms associated with loss of SLC1A4 are similar to, but more severe than, those observed in patients homozygous for missense mutations (Conroy et al., 2016). *In vitro* studies indicated that p.E256K mutant SLC1A4 has a higher affinity for L-serine and L-alanine but a lower maximal transport rate than wildtype SLC1A4 (Damseh et al., 2015). Because serine deficiency disorders caused by a defect in L-serine biosynthesis are also characterized by microcephaly, seizures, and psychomotor retardation, L-serine deficiency is generally considered to underlie the neurological phenotypes of SPACTCCM patients. Since L-serine can be metabolized to D-serine by serine racemase and SLC1A4 can transport both entantiomers (Foster et al., 2016) cognitive impairment in SPATCCM patients may reflect an effect on either L- or D-serine transport, or both.

N-methyl-D-aspartate receptors (NMDARs) play important roles in learning and memory in the mammalian brain (reviewed by Orzylowski et al., 2021; Sherwood et al., 2021). NMDARs are activated by binding of synaptic L-glutamate to GluN2/NR2 subunits and D-serine or glycine to co-agonist sites on GluN1/NR1 subunits. D-serine has three-fold higher affinity for the NMDAR co-agonist site than glycine and acts as a physiological regulator of NMDAR signaling. Dysregulation of D-serine levels at synapses is therefore an important potential factor in neuropathologies arising from NMDA receptor hypo- or hyperfunction. SLC1A4 is thought to play a key role in maintaining neurotransmitter homeostasis and NMDA neurotransmission through regulation of D-serine, but it is not clear whether the phenotypes observed in SPATCCM patients reflect an effect of SLC1A4 mutations on D-serine and NMDA signaling or other roles of this transporter in the CNS.

To better understand the pathogenesis of SPATCCM, we generated and characterized a mouse knock-in model for the p.E256K mutation. We demonstrate that p.E256K mutant SLC1A4 has higher affinity for D-serine but a lower maximum rate of uptake, and mice homozygous for this mutation did not show differences in LTP or performance in behavioral tests that assess learning and memory, although they did show increased anxiety behavior. The corpus callosum and cortical layer 1 of the brains of mice homozygous for the p.E256K mutation are thinner than those of wildtype mice, but no significant difference in myelination was observed. As comprehension and verbal skills are severely impaired in human patients homozygous for the same mutation, our results suggest there may be species differences in the role/importance of SLC1A4 in the CNS.

## Materials And Methods

### Mice

All animal procedures adhered to the US National Research Council’s Guide for the Care and Use of Laboratory Animals, the US Public Health Service’s Policy on Humane Care and Use of Laboratory Animals, and the Guide for the Care and Use of Laboratory Animals, and were approved by the McLaughlin Research Institute’s Institutional Animal Care and Use Committee (IACUC). All mice were housed in the McLaughlin Research Institute’s Animal Resource Center, an all-mouse facility accredited by the American Association for Accreditation of Laboratory Animal Care. Mice were housed in individually ventilated cages under standard conditions, with a 14-hour light/10 hour dark light cycle, and provided Purina 5053 chow and water ad libitum.

*Slc1a4*^*em2Tmg*^ mice, referred to herein as *Slc1a4*^*E256K*^ mutants were generated by CRISPR/Cas9 mediated gene editing in mouse embryos using a single guide RNA (sgRNA), CUUCAAUUCCUUCAAUG (Synthego), and a sense HDR single stranded oligodeoxynucleotide (ssODN) repair template: AGCTAGGCCCCGAGGGAGAAGACCTCATCCGATTCTTCAATTCCTTCAATAAGGCCACCATGGTGCTGGTGTCATGGATCATGTGGTG (Integrated DNA Technologies, Inc.). The G > A mutation at c.978 (the first underlined base) results in p.E256K, while the A > C modification at c.986 (the second underlined base) is a silent change intended to insert an NcoI site to facilitate genotyping, but the founder and pups only showed the c.G978A mutation (second underlined base) that results in p.Glu256Lys. The sgRNA and ssODN were resuspended in embryo microinjection buffer (filter sterilized 5 mM Tris, 0.1 mM EDTA, pH 7.4). The ribonucleoprotein (RNP) mix was prepared by diluting SpCas9 2NLS nuclease (Synthego) and the sgRNA to 4 µM each in Opti-Mem (Gibco) and incubating at room temperature for 10 min prior to adding the ssODN (final concentration of 10 µM). The RNP mix was electroporated into 1-cell mouse embryos following published conditions (Troder et al. 2018), after which embryos were incubated in EmbryoMax Advanced KSOM Embryo Medium (Sigma Cat # MR-101-D) with 3 µM Alt-R HDR Enhancer v.1 (Integrated Data Technologies, Inc.), then transferred to pseudopregnant recipient females at the 1- or 2-cell stage. Founders were identified by PCR and sequencing, as described below, and mated to C57BL/6J mice to identify heterozygotes, which were intercrossed to generate homozygotes. CRISPR founder mice were identified by Sanger sequencing of a PCR product amplified from tail DNA (forward primer: GCTTCCCTGCTGAATCTGAC and reverse primer: ACATGGGAAGGTTGCAAGAC). Sequence data was analyzed using Synthego’s Inference of CRISPR Edits (ICE) tool. Founders were mated to unmanipulated, wildtype C57BL/6J mice and heterozygotes for the edited allele intercrossed. All the animals used in the studies described here were N1F3-N1F6, descended from one male founder. Age-matched wildtype C57BL/6J mice or wildtype siblings from heterozygous intercrosses were used as controls for all studies.

Mice were genotyped either by PCR amplification and sequencing, as described above, or PCR using the following allele-specific primers: wildtype forward CCGATTCTTCAATTCCTTCAATG and reverse atgttttctcctcccaccgt (263 bp product), or mutant forward CCGATTCTTCAATTCCTTCAATA and reverse: ttgatgtgagtccaggggtc (492 bp product). Amplification reactions used Go-Taq Green Master Mix (Promega). Cycling conditions for the wildtype product were: 95 C for 3 min followed by 33 cycles of 95 C for 30 sec, 61 C for 30 sec and 72 C for 60 sec, followed by 3 min at 72 C. Cycling conditions for the mutant product were: 94 C for 3 min followed by 35 cycles of 94 C for 15 sec, 60 C for 30 sec and 72 C for 45 sec, followed by 72 C for 7 min.

### Western Blotting

Sagittal brain hemispheres were homogenized in protein lysis buffer (50 mM Tris, 150 mM NaCl, 1% NP40, 0.1% sodium deoxycholate) supplemented with Complete protease inhibitor cocktail (Roche). Cellular debris was pelleted by centrifugation and the supernatant diluted in 2X SDS loading buffer. Proteins were electrophoresed through 8% SDS-polyacrylamide gels and transferred to Immobilon P membrane (Millipore). Western blotting (WB) was performed following standard protocols using rabbit anti-SLC1A4 (Proteintech Cat# 13067–2-AP, RRID:AB_2190604 or Cell Signaling Technology Cat# 8442, RRID:AB_10828382) and mouse anti-beta-tubulin-III (3F3-G2) (Santa Cruz Biotechnology Cat# sc-53140, RRID:AB_793543). Following ECL (BioRad Clarity ECL substrate), blots were imaged using an Azure 300 imager and quantified using AzureSpot Pro (Azure Biosystems). SLC1A4 expression was normalized to beta-tubulin-III and differences in expression by genotype assessed using a 2-tailed, paired t-test in Microsoft Excel. Similar results were obtained with both SLC1A4 antibodies.

### Histology

Brains were fixed either at room temperature in 10% formalin for at least 1 week or at 4 C in 4% paraformaldehyde for 5 days prior to standard processing and embedding in paraffin. Coronal sections were taken at 5 µm, mounted on positively charged slides, and stained with hematoxylin and eosin (H&E) or processed for immunohistochemical (IHC) staining following standard protocols, using an antibody against Neuronal nuclei (NeuN; Millipore Cat#MAB377, RRID:AB_2298772) at 1:100 or myelin basic protein (MBP; Covance Cat# SMI-99, RRID:AB_2314772) at 1:1000, and DAB chromogen (Biolegend Cat# 926507 and 926606) with manual development to ensure that each negative control remained negative and the positive controls developed signal in the appropriate and expected regions. Slides with no primary antibody were used as negative controls. Brains from wildtype mice were used as positive controls. Images were taken on a Zeiss AxioImagerM1 microscope with a Pixielink A623C color camera and morphometry analyzed using ImageJ and the Fiji image-processing package. Brain structures were measured in millimeters after the settings were established on Fiji. Cortex layer 1 thickness was measured in three separate areas evenly spaced across each field of view. The corpus callosum was measured at the midline. All comparisons between genotypes were made on sections representing similar rostral-caudal regions of the brain.

### Uptake Assays

Xenopus oocytes (EcoCyte Bioscience) injected with approximately 50 ng of human SLC1A4 (wildtype or E256K mutant) cRNA (Ambion mMessage mMachine T7 transcription kit) or uninjected (control) oocytes were incubated with indicated concentrations of [3H]-labeled amino acids (Moravek Biochemicals, Inc.; 20–60 Ci mmol − 1) in Ringer solution (96 mM CaCl, 2 mM KCl, 1 mM MgCl_2_, 1.8 mM CaCl_2_, 5 mM HEPES pH 7.5). Uptake was stopped by washing 3 times with 4°C buffer, then oocytes were lysed in 1.0% sodium dodecylsulfate, and radioactivity was measured by liquid scintillation spectroscopy, as previously described (Foster et al., 2016). D-serine uptake was measured in fresh 300 µm brain slices cut from wildtype and *Slc1a4*^*K/K*^ mice on a VF-200-OZ Compresstome (Precisionary Instruments) in ice-cold sucrose buffer (80 mM NaCl, 75 mM sucrose, 2.5 mM KCl, 1.25 mM NaH_2_PO_4_, 0.5 mM CaCl_2_, MgCl_2_*6H2O, 1 mM ascorbic acid, 3 mM sodium pyruvate, with 5 mM 25 mM glucose and 24 mM NaCO_3_ added immediately before use). Slices were bubbled in artificial cerebrospinal fluid for 15 min, transferred to labeling solution (100 nM ^3^H-D-serine, Moravek Biochemicals, Inc., in 1X aCSF (126 mM NaCl, 2.5 mM KCl, 1.2 mM MgCl_2_*6H2O, 1.2 mM NaH_2_PO_4_, 2.4 mM CaCl_2_ with 11.4 mM glucose and 21.4 mM HEPES)) for 15 min, then washed three times in aCSF. After transferring slices to scintillation vials, 1% SDS was added and samples were left to solubilize overnight before measuring ^3^H activity in a Beckman LS6000IC liquid scintillation counter. Slices were generated from four mice per genotype and four slices were assayed per mouse.

### Acute Brain Slice Recordings

Acute brain slices (300 µm) were sectioned and allowed at least 1 hour to recover at room temperature before being placed in a submersion-type recording chamber perfused at 1.6–2.0 ml/minute with ACSF at 30°C. Slices were visualized on an upright fixed-stage microscope (Olympus BX51WI) equipped with IR-DIC optics. Extracellular field excitatory postsynaptic potentials (fEPSPs) were recorded using glass electrodes filled with ACSF. fEPSPs were induced with 100 µs current pulses between 0.1mA − 0.4mA administered through ACSF-filled stimulating pipettes placed in stratum radiatum. Theta burst stimuli (5 bursts separated by 200 ms of 5 pulses at 100Hz) were administered to induce long-term potentiation. Recordings were made with analog-digital converters and amplifiers from Molecular Devices, and data were acquired at 10kHz and filtered at 2 kHz. Data were acquired and analyzed with pClamp11. Data are presented as mean ± S.E. and were evaluated by Student’s paired t-test or as noted.

### Behavioral Studies

Behavioral studies were performed on three cohorts of 4–5-month-old *Slc1a4*^*K*/K^ homozygotes and wildtype (*Slc1a4*^*E*/E^) controls, all on the C57BL/6J background. The first cohort of 6 males and 5–6 females of each genotype was assessed for open field, novel object, Y-maze, balance beam, and grip strength. The second cohort of 4 wildtype and 6 *Slc1a4*^*K*/K^ males and 5 wildtype and 3–4 *Slc1a4*^*K*/K^ females was assessed for open field, novel object, Barnes maze and grip strength. The third cohort of 7 wildtype and 8 *Slc1a4*^*K*/K^ males and 4 wildtype and 7 *Slc1a4*^*K*/K^ females were subjected to rotarod and grip strength testing. ANY-Maze tracking software (Stoelting Co.) was used for data collection in all studies. Mice were also examined in their home cage for body position, spontaneous activity, tremor, and other general neurobehavioral features.

The novel object tests were performed as described in (Leger et al., 2013) using a 1 day habituation period and white open field as follows. On day 1, mice were allowed to explore the open field freely for 5 min (habituation). For the familiarization stage, 24 h later, two identical objects were placed 5 cm from the walls of the open field and mice were allowed to explore for 10 min. The testing session, where one of the objects was replaced by a new object, occurred 24 h after familiarization and also lasted 10 min. The total number of investigations and time spent investigating each object was recorded for the familiarization and test sessions. An investigation was defined as the mouse being within 2.5 cm of the object, with its nose pointed towards it. Climbing on the object did not count as an investigation.

The Y-maze test was performed according to standard protocols. Briefly, 16 h before the habituation session, mice were singly housed with fresh bedding. The Y-maze was cleaned with 70% ethanol in between mice to minimize odor cues. During the habituation session, mice were introduced to the maze and allowed to explore freely for 5 min, then returned to their home cage for about an hour before the testing session. For the testing session, bedding was placed in each of the arms as follows: clean bedding in the starting arm (arm C), home-cage bedding in one of the short arms (arm A), and bedding from an age- and sex-matched mouse of the other genotype in the remaining arm (arm B), to act as a novel stimulus. Mice were allowed to freely explore for five minutes.

The balance beam test measured time to cross a 1 m long, 6 mm wide beam, and consisted of two training sessions and a test session. The beam and escape box were always cleaned with 70% ethanol between mice. During the training sessions, a mouse was placed on a 12 mm thick beam and gently encouraged to go towards an escape box, which held nesting material from the mouse’s home cage. If a mouse stopped moving during the run, a gentle tap on the back was given to encourage forward progress. If a mouse refused to go across the beam, the researcher held the mouse by the tail and nudged it toward the escape box. Timing started when the hind legs crossed the start line and ended when the hind legs crossed the finish line. Mice were allowed a brief rest (approx. 15 sec) in the escape box before being repositioned at the start line to repeat the trial. After 3 trials, the mouse was returned to its home cage for a 10-minute break before repeating 3 training runs on a 6 mm wide beam. This entire process was repeated 24 hours after the first training session. The testing session consisted of three runs across the 6 mm beam 24 h after the second training session.

The Barnes maze test included 1 habituation session, 8 spatial acquisition sessions, and 1 probe session. First, mice were placed on the Barnes maze and allowed to explore freely for 120 sec (habituation). Solid black cues (a triangle, circle, square, and lightning bolt) were placed on the four walls around the maze to provide spatial orientation. At the end of habituation, a clear beaker was placed over the mouse to guide it to the escape hole, where it was kept for 120 sec. The spatial acquisition session took place 24 h after habituation. The target hole and escape tunnel were moved 180 degrees from their original position. Mice were placed under a start cup in the middle of the maze and aversive stimuli (bright lights and 80-decibel hairdryer audio) were used to encourage the mice to find the target hole as quickly as possible. Mice were released from the start box and allowed to roam freely until they found and entered the target hole, or until 3 min had passed; if the mice did not find the target hole within 3 min, they were guided to the target hole and allowed to remain there for about 30 sec. Two spatial acquisitions were done per day for four days. For the probe trial, which took place 72 h after the last spatial acquisition session, the escape chamber was closed off. As in the training sessions, the mice were placed under a start box and then allowed to explore the maze freely for 90 sec. Aversive stimuli were present during the probe trial.

Grip Strength was assessed using a Bioseb-GS3 Grip Strength Meter. Mice were held over the grid until they grasped it with only their forepaws, then gently tugged by the tail until they released the grid and maximal peak force recorded. The same process was repeated with all four paws engaging the grid.

For the Rotarod test, mice from the same cage were placed on the apparatus (Maze Engineers) facing forward at a speed of 4 rpm. Once the mice were in placed, the Rotarod accelerated from 4 to 40 rpm over 300 sec. The latency for the mice to fall was recorded. This was done three times a day with at least 15 min in between trials, for three consecutive days.

### Statistical Analyses

Unless otherwise indicated, data were analyzed using GraphPad Prism 9. For behavioral studies, differences by genotype were assessed by two-tailed, paired or unpaired T-tests with Welch’s correction, except rotarod data, which was assessed using two-way ANOVA for average latency and distance and linear regression for drop speed. Data for males and females of the same genotype were first assessed separately, then combined if no significant difference was observed.

## Results

The goals of the studies described here were to determine the effect of a recessive *SLC1A4* mutation associated with a human neurodevelopmental disorder on D-serine uptake and to generate and characterize a knock-in mouse model. Several different *SLC1A4* mutations have been described in patients with SPATCCM. As the p.E256K allele originally identified in the Ashkenazi Jewish population has also been detected in Hispanic and South Asian populations Conroy et al., 2016), we focused on the impact of this mutation.

**Figure 1:**
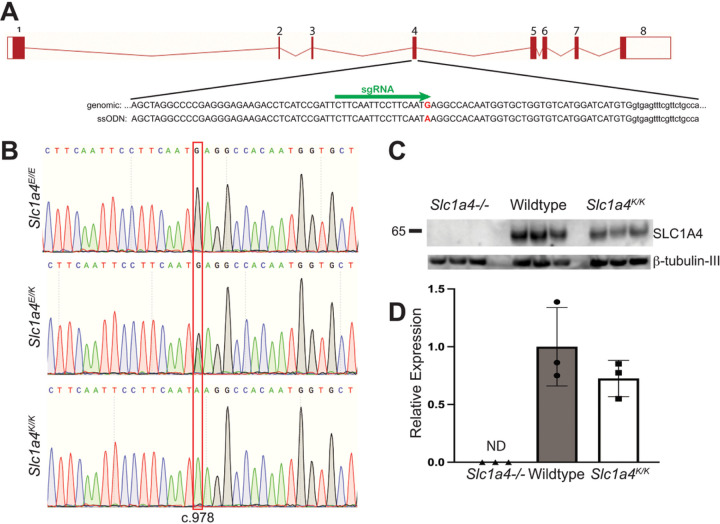
Generation & characterization of *Slc1a4*^*E256K*^ (*Slc1a4*^*K/K*^) mutant mice. (A) Schematic of the Slc1a4 locus, showing sequence of exon 4 in the region targeted by CRISPR/Cas9 gene editing. The sgRNA binding site is indicated by the green arrow and the cytosine mutated to adenine is shown in red. (B) Chromatograms from Sanger sequencing of PCR products spanning the region targeted by CRISPR/Cas9 gene editing from wildtype (top), heterozygous (middle) and homozygous mutant (bottom) mice showing the G to C change corresponding to position 978 of the cDNA sequence. (C-D) Western blot analysis showed similar expression of SLC1A4 in the brains of wildtype (*Slc1a4*^*E/E*^) and *Slc1a4*^*K/K*^ mutant mice. Relative expression of SLC1A4, normalized to b-tubulin-III. SLC1A4 expression was not significantly different between wildtype and *Slc1a4*^*K/K*^ mutant mice, and was not detected (ND) in the null mutants.

Mice carrying the c.G978A mutation that alters the glutamate at position 256 to a lysine (p.E256K) were generated by CRISPR/Cas9-mediated gene editing (Fig. 1A-B). Two founders carrying the desired edited allele (c.G978A) were obtained in one experiment: a male carrying the edited allele at a frequency of ~ 28% and a female founder carrying it at ~ 15%. Both transmitted the mutation to their offspring and homozygotes were obtained at Mendelian frequencies from intercrosses between heterozygotes. Homozygotes were viable, fertile, and grossly normal. All mice used in the studies described here descended from the male founder. To assess whether the p.E256K mutation altered SLC1A4 expression, brain protein lysates from 3-month-old C57BL/6J-*Slc1a4*^*K/K*^ and wildtype (*Slc1a4*^*E/E*^) controls were subjected to western blotting with an antibody against SLC1A4. Brain lysates from *Slc1a4* null mutants, included to verify specificity of the antibody, showed no detectable SLC1A4. No significant difference in SLC1A4 levels was observed between *Slc1a4*^*K/K*^ and wildtype brains when normalized to β-tubulin-III (Fig. 1C-D).

**Figure 2:**
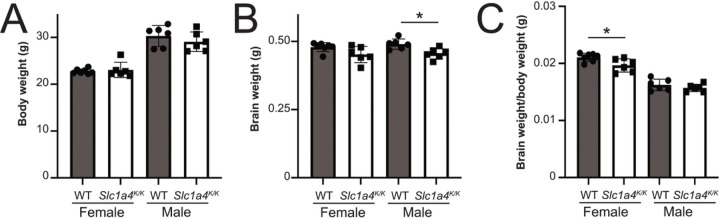
Body and brain weight in *Slc1a4*^*K/K*^ mutants. (A) No significant differences were observed in body weight between *Slc1a4*^*K/K*^ and wildtype males or females. (B) Mutant males had significantly reduced brain weight; *p=0.01. (C) Mutant females had a significantly lower brain weight to body weight ratio than controls; *p=0.02.

Human patients homozygous for the p.E256K mutation have microcephaly, developmental delay, and seizures. *Slc1a4*^*K/K*^ mutant mice did not show any overt phenotypes and were never observed to have seizures, even upon handling, nor were they prone to sudden, unexplained death. We assessed brain and body weight of a cohort of 3-month-old wildtype and *Slc1a4*^*K/K*^ males and females (n = 6–7 per group). There were no differences in body weight (Fig. 2A). Relative to wildtype mice, brain weight was significantly lower in *Slc1a4*^*K/K*^ males, while the difference in females was suggestive but not significant (p = 0.06; Fig. 2B). When normalized to body weight, the difference in males (3% lower than wildtype) was no longer significant but the difference in females was (7% lower than wildtype; Fig. 2C).

**Figure 3:**
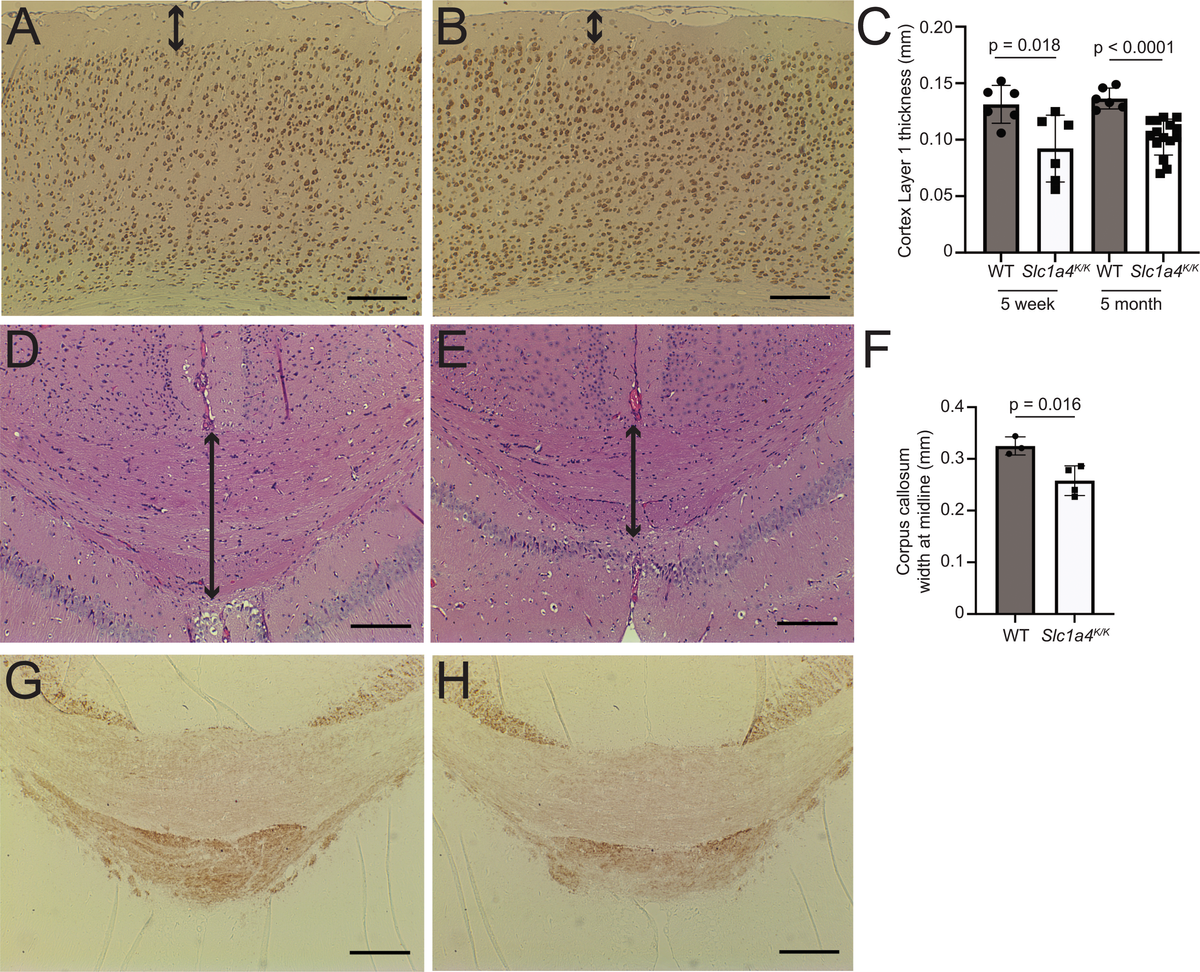
Histopathological analysis of *Slc1a4*^*K/K*^ brains. Coronal brain sections from 5-month-old wildtype (A,D,G) and *Slc1a4*^*K/K*^ (B,E,H) mice were stained for NeuN (A,B), hematoxylin and eosin (D,E) and MBP (G,H) to assess morphology and myelination. Cortex layer 1 (arrows in A-B, quantified in C) and the corpus callosum (arrows in D-E, quantified in F) were significantly thinner in *Slc1a4*^*K/K*^ mutant brains, but the level and distribution of MBP in the corpus callosum was similar to wildtype (G-H). Scale bars: 200 mm.

The brains of SPATCCM patients homozygous for the p.E256K mutation show hypomyelination and a thin corpus callosum. Histopathological analysis of *Slc1a4*^*K/K*^ and control brains at 5 weeks and 5 months of age revealed a significant reduction in the thickness of layer 1 of the cortex (Fig. 3A-C) as well as thinning of the corpus callosum at the midline (Fig. 3D-F). IHC for myelin basic protein (MBP) showed a similar pattern and intensity of staining between wildtype and *Slc1a4*^*K/K*^ brains in the corpus callosum (Fig. 3G-H) and other brain regions (data not shown).

**Figure 4:**
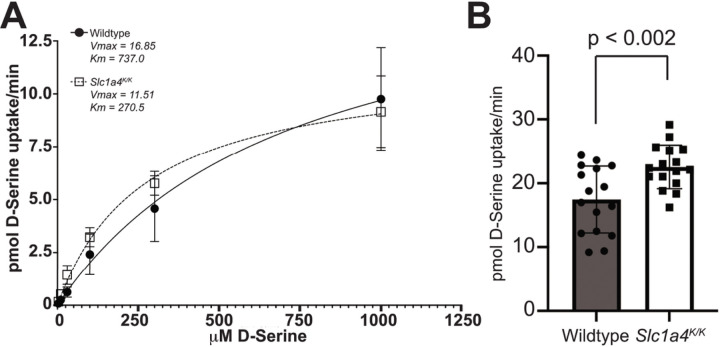
The p.E256K mutation alters the kinetics of D-serine uptake. (A) Wildtype or p.E256K mutant human SLC1A4 was expressed in Xenopus oocytes and [3H]-labeled D-serine uptake measured by liquid scintillation spectroscopy. The mutant transporter consistently showed increased affinity for D-serine (Km) but lower maximum velocity (Vmax), indicating saturation at a lower concentration of D-serine. (B) Brain slices from *Slc1a4*^*K/K*^ mice showed higher [3H]-labeled D-serine (100 nM) uptake than *Slc1a4*^*E/E*^ (wildtype) brain slices, consistent with the mutant transporter change in D-serine kinetic parameters.

It was previously reported that p.E256K mutant SLC1A4 displays higher L-serine and -alanine affinity and lower maximal transport rates, with similar substrate selectivity as wildtype SLC1A4 in a heterologous system (Damseh et al., 2015). As SLC1A4 has more recently been shown to bind D-serine (Foster et al., 2016), we examined the kinetics of SLC1A4 p.E256K transport of D-serine. Expressing p.E256K and wildtype SLC1A4 in frog oocytes, we consistently observed increased affinity of the mutant transporter for D-serine, but a lower maximum velocity (Fig. 4A).Consistent with these data from exogenously expressed SLC1A4 E256K, 300 µm thick acute brain slices from *Slc1a4*^*K/K*^ mice showed increased uptake of 100 nMD-serine relative to slices from *Slc1a4*^*E/E*^ (wildtype) mice (Fig. 4B), suggesting that the mutant protein transports normal physiological concentrations of D-serine at an increased rate.

**Figure 5:**
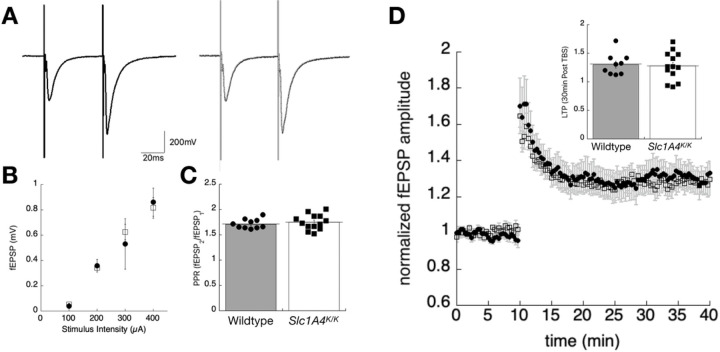
Extracellular field potentials measured from the stratum radiatum of the hippocampus in *Slc1a4*^*K/K*^ and wildtype mice. (A) Sample traces showing representative field responses in hippocampal slices from wildtype (left, dark traces) or *Slc1a4*^*K/K*^ (right, light traces) mice, evoked by paired stimuli in stratum radiatum (50 ms interpulse interval). (B) Input/output relationship, showing the peak amplitude of the first EPSP plotted as a function of stimulus strength. (C) The average paired pulse facilitation ratio, taken as the peak amplitude-2/amplitude-1, shows no statistically significant differences in this form of short facilitation. (D) Synaptic plasticity was assessed by theta burst stimulation (TBS 1x) in the stratum radiatum. The time series measuring the first pea amplitude shows a 10-minute baseline, then enhanced synaptic response following the TBS. The inset shows the average long-term potentiation (LTP) taken 30-min post-TBS stimulation for the two genotypes.

Altered D-serine homeostasis in the brain could affect NMDAR signaling and have an impact on learning and memory. Consistent with this, human SPATCC patients homozygous for the p.E256K mutation show reduced comprehension and verbal skills. To assess synaptic transmission in the mice, extracellular field potentials were recorded in the CA1 stratum radiatum of wildtype and *Slc1a4*^*K/K*^ mice (Fig. 5A-C). Paired stimuli were given 50 ms apart and no differences in amplitude, rise, or decay of the field EPSPs were measured (Fig. 5A). An input/output relationship was established by plotting the peak amplitude achieved with increasing stimulus strength between (0–400uA) (Fig. 5B). The short-term facilitation induced by paired-pulse stimulation was not statistically different between genotypes (wildtype facilitation ratio, 1.71 +/−.03; *Slc1a4*^*K/K*^ facilitation ratio1.75 +/− .04;Fig. 5C). Synaptic plasticity at this Shaffer-CA1 pyramidal cell synapse was assessed by stimulating in stratum radiatum with a theta burst stimulation protocol to induce long-term potentiation (LTP). The average potentiation in amplitude 30min post-TBS showed no differences between wildtype (1.3 +/− .06) and *Slc1a4*^*K/K*^ (1.28 +/− .06) mice (Fig. 5D).

**Figure 6:**
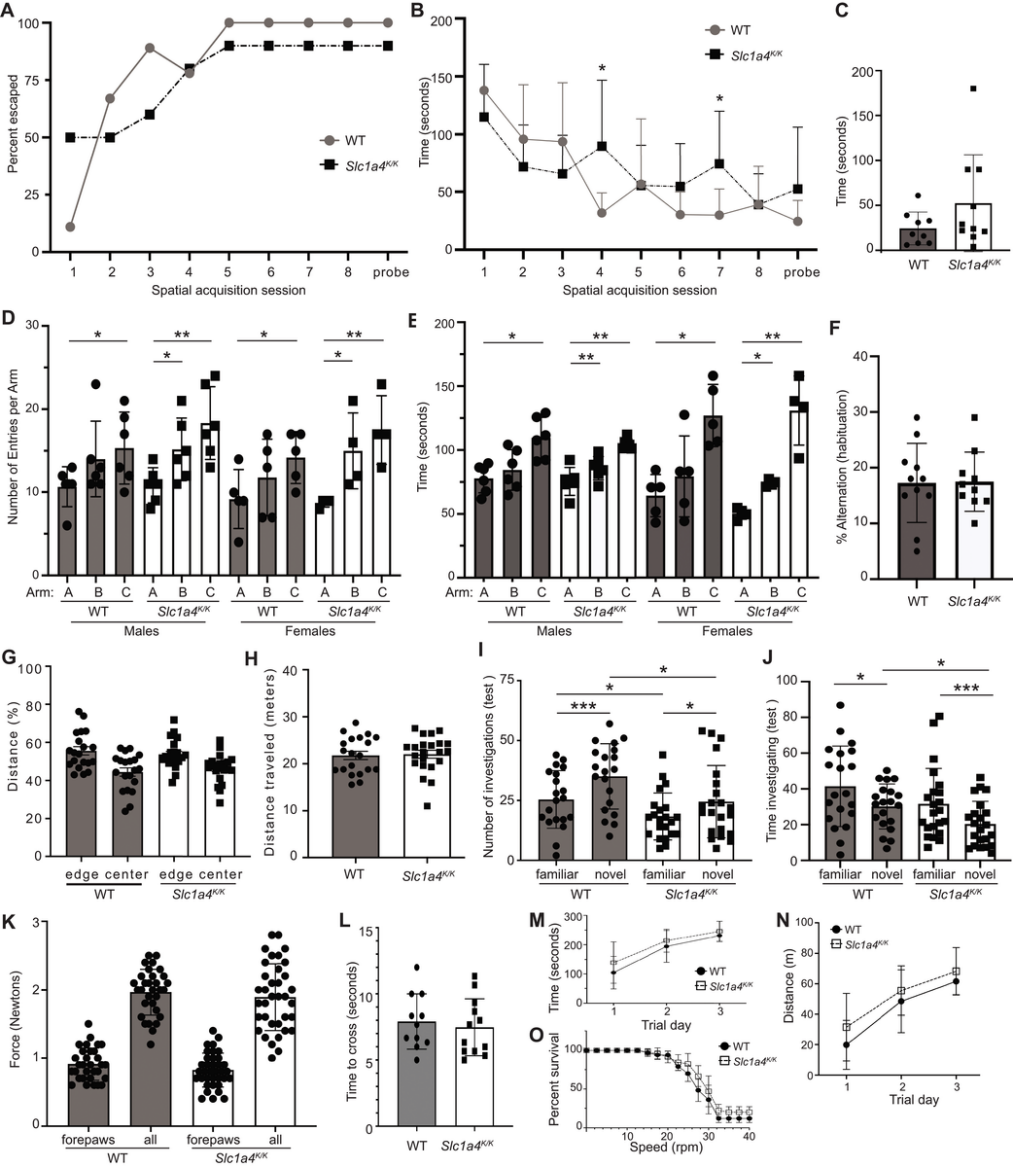
Neurobehavioral testing was performed to further assess learning and memory, as well as motor coordination, in *Slc1a4*^*K/K*^ mice. (A-C) The Barnes maze assesses spatial learning and memory. There were no significant differences in performance between 5-month-old wildtype and *Slc1a4*^*K/K*^ mice. (D-F) The Y-maze assesses social exploratory behavior. Home-cage bedding was placed in arm A, bedding from a foreign cage in arm B, and unused bedding in arm C. All animals, regardless of sex or genotype, spent the least amount of time in and had fewest entries into arm A, and spent the most time in and had the most entries into arm C (D-E). While there was a non-significant trend for wildtype mice, *Slc1a4*^*K/K*^ mice of both sexes showed a significant increase in entries into and time spent in arm B than arm A. There was no significant difference in percent alternation (a measure of spatial working memory) between wildtype and mutant animals (F). (G-H) There were no significant differences in time spent in the center or edges (G) or total distance traveled (H) in the Open Field test, indicating no gross motor defect and normal exploratory behavior. (I-J) The Novel Object Recognition test evaluates cognition, particularly recognition memory. Wildtype and mutant mice explored the novel object more often but for less time than the familiar object, but the number of investigations of either object was significantly lower by *Slc1a4*^*K/K*^ mutants and they spent significantly less time investigating the novel object, as compared to wildtype mice (I,J). (K) Grip strength was measured to assess neuromuscular function based on maximal muscle strength. No significant differences were observed for forelimbs alone or all limbs combined. (L-O) No significant differences were observed for time to cross a balance beam (L) or in rotarod performance (M: average latency; N: percent survival (percent of mice for each genotype still on the rotarod at given speed); O: distance traveled). ***p<0.001; **p<0.01,*p<0.05.

To evaluate the impact of the p.E256K mutation on behaviors, we subjected *Slc1a4*^*K/K*^ mice to a battery of tests to assess learning, memory, anxiety, locomotion, strength, balance, motor coordination, and endurance. The Barnes maze assesses spatial learning and memory. Consistent with the lack of difference in hippocampal LTP between wildtype and *Slc1a4*^*K/K*^ mice, there were no significant differences in time to escape or escape percentage in the Barnes maze test of spatial learning and memory (Fig. 6A-C). The Y maze was used to assess social exploratory behavior. All animals, regardless of sex or genotype, spent the least amount of time in and had the fewest entries into arm A, containing their home cage bedding, and spent the most time in and had the most entries into arm C, containing fresh bedding (Fig. 6D-F). While there was a non-significant trend for wildtype mice, *Slc1a4*^*K/K*^ mice of both sexes did show a significant increase in entries into and time spent in arm B (bedding from an unfamiliar cage) than arm A (home cage bedding).

The open field test assessment of locomotor activity, anxiety, and exploratory behaviors showed that both wildtype and *Slc1a4*^*K/K*^ mice spent significantly more time at the edges than in the center (p < 0.002), and there were no significant differences between genotypes in total distance traveled or time spent in the center or edges (Fig. 6G-H and data not shown), indicating that the mutants have normal locomotor activity and aversion to bright light and/or open spaces. The novel object test assesses learning and memory as well as exploratory behavior and anxiety. Wildtype and *Slc1a4*^*K/K*^ mice investigated the novel object more frequently but for less time, but the number of times *Slc1a4*^*K/K*^ mice investigated either object was significantly lower than controls, and the mutants spent significantly less time investigating the novel object compared to wildtype mice (Fig. 6I-J).

As human p.E256K patients show spasticity, we used the grip strength test to measure neuromuscular function based on maximal muscle strength of forelimbs alone and fore- and hind-limbs combined. Data from males and females were combined since they did not show significant differences in either genotypic group (p > 0.22). Grip strength for forepaws only and all paws combined showed no significantly differences between *Slc1a4*^*K/K*^ and wildtype mice (p > 0.10; Fig. 6K). Consistent with this finding, *Slc1a4*^*K/K*^ mice did not show a significant difference in time to cross a 6-mm-wide balance beam (p > 0.60; Fig. 6L). Wildtype and *Slc1a4*^*K/K*^ mice also showed no significant differences in performance on the rotarod (Fig. 6M-O). Wildtype and mutant mice both stayed on the rotarod longer and went further each day of testing (M,N) and they dropped off at a similar rate as rotarod speed increased (shown as percent survival in Fig. 6O).

## Discussion

It has generally been assumed that the phenotypes of SPATCCM patients are a result of disrupted L-serine transport, as they overlap with those of serine deficiency disorders (psychomotor retardation, microcephaly and seizures) (Heimer et al., 2015; Damseh et al., 2015; Srour et al., 2015). L-serine plays an essential role in numerous cellular pathways, including protein synthesis, neurotransmission, and synthesis of sphingolipids, phospholipids, L-cysteine, phosphatidyl-L-serine, and D-serine, all of which have important roles in the brain (El-Hattab, 2016; Grant, 2018). The recent identification of D-serine as a substrate for transport by SLC1A4 raises the possibility that cognitive impairment in SPATCCM patients reflects disrupted NMDAR signaling [2]. A mouse model of SPATCCM would be a valuable resource for dissecting disease pathogenesis and identifying therapeutic strategies. We therefore used CRISPR/Cas9 gene editing to introduce a c.G978A mutation to alter the glutamate at position 256 to a lysine (p.E256K), replicating a disease-associated mutation found in human SPATCCM patients.

The p.E256K mutation in SLC1A4 was previously shown to reduce maximal rates of L-serine and L-alanine uptake by 50–60%, but to significantly increase the transporter’s apparent affinity (by 2–3X) for those amino acids (Damseh et al., 2015). Analyzing D-serine uptake in frog oocytes expressing wildtype and p.E256K SLC1A4 and in brain slices from wildtype and *Slc1a4*^*K/K*^ mutant mice, we demonstrate that p.E256K SLC1A4 also has a higher affinity and reduced Vmax for D-serine. While equal amounts of cRNA were transcribed and injected from wild -type and mutant cDNAs that were identical except for the point mutation, a definitive quantitative comparison of Vmax values cannot be made. Nevertheless the overall kinetic changes seen here with D-serine are consistent with those reported for L-serine by Damseh et al. (2015). This indicates that at low D-serine concentrations, p.E256K likely acts as a gain-of-function mutation, but at high concentrations of D-serine, it would act as a loss-of-function mutation.

*Slc1a4* ^*K/K*^ mice had neuroanatomical phenotypes consistent with those observed in SPATCCM patients, including thinning of the corpus callosum and microcephaly. They also showed a significant reduction in the thickness of cortical layer 1, but no obvious hypomyelination. *Slc1a4*^*K/K*^ mice were not seizure-prone, but the fact that not all human patients develop seizures suggests this phenotype may be influenced by genetic or environmental modifiers. SPATCCM patients are typically hypotonic, but strength, locomotor activity, balance, coordination and endurance of in *Slc1a4*^*K/K*^ mice showed no significant differences from wildtype, as assessed by the grip strength, open field, balance beam, and rotarod tests. The results of the novel object recognition test indicated that *Slc1a4*^*K/K*^ mice can distinguish novel and familiar objects, consistent with their performance in the Barnes maze and lack of an LTP phenotype; however, the mutants investigated the novel and familiar objects significantly less than wildtype mice, despite not showing any deficits in exploratory behavior in the Y maze. The Y-maze was primarily an olfactory-based paradigm, and also assessed social behavior, while the novel object recognition test was more visual, suggesting there may be differences in how *Slc1a4*^*K/K*^ mutant mice process and interpret different environmental cues. Altered processing of visual information would be consistent with the significant reduction in the volume of cortex layer 1 observed in the mutants.

Mutations in *SLC1A4* predicted to result in complete loss-of-function are associated with a more severe phenotype in human patients. Mice homozygous for a loss-of-function mutation (*Scl1a4*^*tm1e*^) were reported to have decreased hippocampal and striatal volume, lower activity in the open field, and modestly impaired spatial acquisition and spatial learning reversal in the Morris water maze (Kaplan et al., 2018). We did not see a significant difference in spatial acquisition for *Slc1a4*^*K/K*^ mutants in the Barnes maze. Similar to what we observed for *Slc1a4*^*K/K*^ mutants, synaptic plasticity was normal in mice homozygous for the *Scl1a4*^*tm1e*^ allele. Taken together, these results indicate that there are significant species differences in the effects of both loss-of-function and gain-of-function SLC1A4 mutations, with generally less severe neurophysiological and behavioral phenotypes seen in *Slc1a4* mutant mice. This could suggest that compensatory or redundant mechanisms are present in murine brain that control homeostasis of L-serine, D-serine, and possibly other amino acid substrates of SLC1A4. Studies to understand the mechanism underlying these species-specific effects could identify novel strategies to treat SPATCCM.
